# *RE*BCO mixtures with large difference in rare-earth ion size: superconducting properties of chemical solution deposition-grown Yb_1−*x*_Sm*_x_*Ba_2_Cu_3_O_7−_*_δ_* films

**DOI:** 10.1098/rsos.201257

**Published:** 2020-11-18

**Authors:** Pablo Cayado, Minjuan Li, Manuela Erbe, Zhiyong Liu, Chuanbing Cai, Jens Hänisch, Bernhard Holzapfel

**Affiliations:** 1 Karlsruhe Institute of Technology (KIT), Institute for Technical Physics (ITEP), Hermann-von-Helmholtz-Platz 1, 76344 Eggenstein-Leopoldshafen, Germany; 2 Shanghai Key Laboratory of High Temperature Superconductors, Physics Department, Shanghai University, Shanghai 200444, People's Republic of China

**Keywords:** YbSmBCO, chemical solution deposition, extremely low fluorine, rare-earth ion size, pinning performance

## Abstract

The main objective of this work was to study the superconducting properties of *RE*BCO films with a mixture of rare-earth (*RE*) ions with large difference in ion size, in particular Sm^3+^ and Yb^3+^. These Yb_1−*x*_Sm*_x_*Ba_2_Cu_3_O_7−_*_δ_* films have been successfully prepared for the first time by chemical solution deposition following the extremely low-fluorine route, which allows reducing the fluorine content by 93% with respect to standard full trifluoroacetate solutions. On the one hand, critical temperature *T*_c_ remains stable at approximately 90 K with Sm content up to *x* = 0.5 where *T*_c_ starts to increase towards the values of pure SmBCO films of approximately 95 K. On the other hand, the critical current densities *J*_c_ of the pure SmBCO films are the largest at 77 K, where the influence of a higher *T*_c_ is very relevant, while at lower temperatures and low fields, the mixed films reach larger values. This demonstrates that mixing rare-earth elements *RE* in *RE*Ba_2_Cu_3_O_7−_*_δ_* causes a change in the pinning properties of the films and reveals the importance of selecting adequate *RE*BCO compounds according to the temperature and magnetic field region of a desired application.

## 1. Introduction

In recent years, the second-generation (2G) high-*T*_c_ superconducting tapes, the coated conductors (CCs) have captured large interest in applied superconductivity [1–3]. Their high critical current densities, also in high magnetic fields, make them very attractive for numerous applications such as high-field magnets, motors, generators and fault-current limiters. However, in order to spread their use in those applications, the currently available CCs still require an improvement of their performance, i.e. the superconducting properties of the *RE*Ba_2_Cu_3_O_7−_*_δ_* (*RE*BCO) films within the CC's architecture need to be enhanced.

One of the options for achieving such improvements is the use of alternative *RE*BCO compounds, i.e. compounds with rare-earth (*RE*) ions other than Y^3+^, which forms YBa_2_Cu_3_O_7−_*_δ_* (YBCO), the best-studied *RE*BCO compound. For some of those alternative cases, improved superconducting properties compared to YBCO have been reported [4–8]. However, the synthesis of these compounds is occasionally more complicated than for YBCO. On the one hand, large *RE* ions, like Nd^3+^ or Sm^3+^, tend to partially replace the Ba^2+^ ions and, on the other hand, small *RE*^3+^ ions, such as Yb^3+^ and Lu^3+^, do not fit satisfactorily in their lattice site, generating vacancies. Both facts cause a drastic decrease in the *RE*BCO phase stability and superconducting properties [9–11]. In particular, the syntheses of SmBCO and YbBCO films are probably two of the most challenging ones among the *RE*BCO phases because the ion size of both compounds are almost at the superior and inferior ends of the line of possible lanthanide candidates. Recently, our group has been able to prepare SmBCO films by chemical route showing the difficulties associated with the tendency of Sm^3+^ ions to occupy the Ba^2+^ sites [12]. Also, several works reported the problems in preparing YbBCO films, leading, in general, to quite modest maximum critical temperatures *T*_c_ ∼ 90 K and self-field critical current densities, *J*_c_^sf^, at 77 K of approximately 1 MA cm^−2^ [13–18].

Another approach, and probably the most important one, for improving the superconducting properties of *RE*BCO films is to increase the flux pinning. Besides the usual strategy of preparing nanocomposites, e.g. by adding non-superconducting perovskite nanoparticles, several studies reported on possible flux pinning enhancement in *RE* mixture compositions with respect to the standard pure *RE*BCO compounds [19–21]. The *RE* elements involved in these mixed compounds and their ratio have to be chosen wisely. The optimum crystallization temperatures of the different *RE*BCO phases depend on the *RE*: they increase continuously from the *RE*BCO compounds with smaller ions (YbBCO, ErBCO, YBCO) to those with bigger ions (EuBCO, SmBCO, NdBCO) reaching even more than 100°C of difference [22,23]. Therefore, the synthesis of mixed compounds with a large difference in *RE* ion size may be more difficult and require a compromise between two optimal temperatures that might be more than 100°C apart.

The chosen technique to prepare the films for this study is the low-cost, versatile and easy-to-scale chemical solution deposition (CSD). Most of the works using this technique are based on the well-known trifluoroacetate–metal organic decomposition (TFA-MOD) route [24], in which the fluorine-containing precursor solutions decompose in the pyrolysis to metal fluorides avoiding the undesirable formation of BaCO_3_. This compound is very difficult to decompose in the subsequent crystallization stage since much larger temperatures would be needed than used in the crystallization process [25]. However, despite the advances in recent years, the TFA-MOD route still needs quite long times to obtain defect-free pyrolysed films [7,26–28], and to shorten this process would be desirable to increase the production rates.

The time restrictions associated with the pyrolysis in the TFA-MOD route can be overcome by changing the solution formulation towards lower contents of fluorinated precursors [29–31]. For YBCO films, Li *et al.* [32] recently tested several precursor solutions with different fluorine contents: the conventional low-fluorine solution with a fluorine content of 54%, the super low-fluorine solution with a fluorine content of 31% and the extremely low-fluorine (ELF) solution with a fluorine content of only 7% with respect to the full trifluoroacetates (TFA) solution. All these low-fluorine solutions still follow the so-called BaF_2_ reaction mechanism for the formation of *RE*BCO films, which is a well-understood process and much easier to handle than the alternative fluorine-free process. A big advantage of a decreasing fluorine content in the precursor solutions is the possibility to increase the heating rate of the pyrolysis for YBCO films. In the particular case of the ELF solutions, excellent superconducting properties of YBCO films have been achieved using much faster heating rates than with other types of solutions. However, the ELF solutions have not been investigated for the synthesis of other *RE*BCO compounds apart from our recent work on SmBCO films [12]. Here, we extend those investigations on ELF *RE*BCO films towards mixed *RE* films, especially Yb_1−*x*_Sm*_x_*Ba_2_Cu_3_O_7−*δ*_ (YbSmBCO) containing *RE* ions with very small (Yb) and very large (Sm) diameter.

## 2. Experimental procedure

### 2.1. Solution and thin film preparation

The YbSmBCO-ELF solutions were prepared following the procedure explained in detail in [12]: Yb, Sm and Cu acetates (purity > 99.99%, Alfa Aesar) were dissolved in the desired stoichiometry in propionic acid (99%). Concerning Ba, 1/3 of the Ba acetate was dissolved in trifluoroacetic acid and deionized water to convert it to TFA while the remaining 2/3 were also dissolved in propionic acid together with the other acetates. Afterwards, the two solutions were dried in a rotary evaporator, and the resulting highly viscous residues were re-diluted in anhydrous methanol (99.9%). After an iterative purification process, both solutions were mixed, and the final concentration of 2 mol l^−1^ (sum of total metal concentration) was adjusted by adding or evaporating methanol. With this procedure, an F/Ba ratio of 2 was achieved, which is the minimum amount of F necessary to allow a full conversion of the Ba precursor to BaF_2_ avoiding the detrimental BaCO_3_ formation [29].

The final solutions with different Yb/Sm compositions were deposited on (100)-oriented LaAlO_3_ (LAO) single crystal substrates via spin coating (6000 r.p.m., 2 min). The as-deposited films were pyrolysed and grown with the thermal profiles and gas mixture given in [12]: heating with 20°C min^−1^ in a flow of 2 l min^−1^ of pure O_2_ for the pyrolysis at 500°C, and heating with 20°C min^−1^ in 2 l min^−1^ of an N_2_/O_2_ mixture with 50–200 ppm O_2_ for the growth at 720–820°C. The pyrolysis parameters were the same for all solutions while crystallization temperature, *T*_crys_, and oxygen partial pressure, *p*O_2_, had to be adjusted in the given ranges for each composition.

### 2.2. Thin film characterization

The critical temperature *T*_c_ (*T*_c,90_, i.e. the temperature of 90% normal state resistance above the transition), Δ*T*_c_ (*T*_c,90_ – *T*_c,10_), *J*_c_(*B*) (via voltage-current *V*(*I*) curves with 1 µV cm^−1^ criterion) and pinning force density *F*_p_ = *J*_c_ ⋅ *B* of the films were studied via transport measurements in four-point geometry on a 14 T Quantum Design physical property measurement system (PPMS). The measurements were done on 10–20 µm wide tracks prepared by photolithography and wet-chemical etching. The dimensions of the tracks were determined by a Bruker Dimension Edge atomic force microscope (AFM). The average thickness of all films in this work was approximately 270 nm.

## 3. Results and discussion

### 3.1. Growth process of Yb_1−*x*_Sm*_x_*Ba_2_Cu_3_O_7−_*_δ_* films

**Figure 1.**
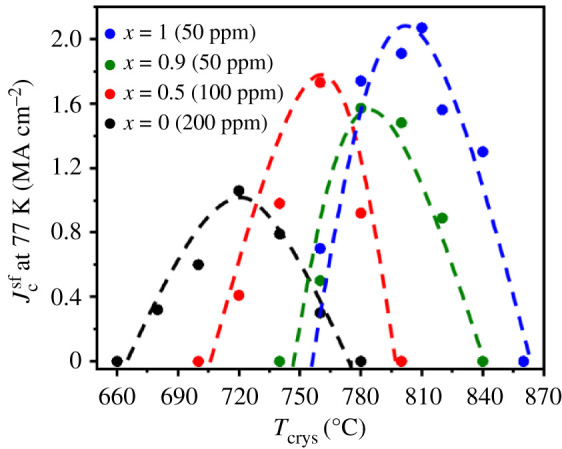
Dependence of $J_{c}^{\mathrm{sf}}$ at 77 K on *T*_crys_ for Yb_1−*x*_Sm*_x_*Ba_2_Cu_3_O_7−_*_δ_* films with *x* = 0, 0.5, 0.9 and 1 at their optimal *p*O_2_ value. The dashed lines are guides for the eyes.

The pyrolysis with a heating rate of 20°C min^−1^ led to homogeneous, smooth and defect-free films confirming the results of [12]. As reported in [21], the crystallization temperature, *T*_crys_, and the oxygen partial pressure, *p*O_2_, need to be adjusted for each composition since they depend strongly on the *RE* ion size. This optimization for each Sm content *x* was carried out following the strategy of [21]: varying *T*_crys_ and *p*O_2_ independently by keeping the respective other parameter constant while searching for the highest possible $J_{c}^{\mathrm{sf}}$ at 77 K and so determining iteratively the most suitable combination of *T*_crys_ and *p*O_2_ for each compound. Figure 1 shows the dependency of $J_{c}^{\mathrm{sf}}$ at 77 K on *T*_crys_ for a constant, optimal *p*O_2_ for each composition. The optimal *T*_crys_ of the pure YbBCO (approx. 720°C) and SmBCO (approx. 810°C) films differ by 90°C due to the large difference in *RE* ion size. The optimal *T*_crys_ for the mixed compounds was searched for by exploring the temperature range spanned by the pure phases. While the maxima lie between the optima of the pure phases as expected, the mixed compounds surprisingly show a narrower *T*_crys_ range for reasonable $J_{c}^{\mathrm{sf}}$ values at 77 K than the single-phase compounds. This suggests that the formation of mixed *RE*BCO compounds with very different *RE* ion sizes is rather complex and requires specific growth conditions to reach reasonable superconducting properties. This is strikingly different to our recent work on full-TFA grown mixed *RE* films with *RE* sizes not too far apart [23], which presented smoother and even wider temperature windows than the single-phase compounds. The optimal *p*O_2_ values follow the same trend as the ones reported in [21] with lower values for the compounds with bigger *RE*^3+^ ion size, i.e. for larger *x* values. Table 1 summarizes the most suitable parameters *T*_crys_ and *p*O_2_ for the Sm contents *x* in our study as well as the resulting physical properties.

**Table 1.** Optimal crystallization temperature (*T*_crys_) and oxygen partial pressure (*p*O_2_) for the growth of Yb_1−*x*_Sm*_x_*Ba_2_Cu_3_O_7−_*_δ_* films and the superconducting properties of films grown at these conditions.

*x*	*T*_crys_ (°C)	*p*O_2_ (ppm)	*T*_c_ (K)	Δ*T* (K)	$J_{c}^{\mathrm{sf}}$ 77 K (MA cm^−2^)	$J_{c}^{\mathrm{sf}}$ 65 K (MA cm^−2^)	$J_{c}^{\mathrm{sf}}$ 30 K (MA cm^−2^)	H* 77 K (mT)	H* 65 K (mT)	H* 30 K (mT)
0	720	200	90.5	1.1	1.1	2.0	3.9	8	17	29
0.5	760	100	91.1	1.3	1.7	2.9	4.8	13	32	44
0.9	780	50	93.9	1	1.6	2.9	6.1	10	47	56
1	810	50	95.0	0.9	2.1	3.0	4.7	11	20	34

### 3.2. Superconducting properties of Yb_1−*x*_Sm*_x_*Ba_2_Cu_3_O_7−_*_δ_* films

The Yb-containing films present similar microstructural characteristics to the SmBCO films in [12]: a strong preference for the *c*-axis orientation together with occasionally surfacing misoriented grains. This has been the first time that ELF solutions were used to successfully grow this type of films, and one of the first to study this *RE* combination at all [33].

**Figure 2.**
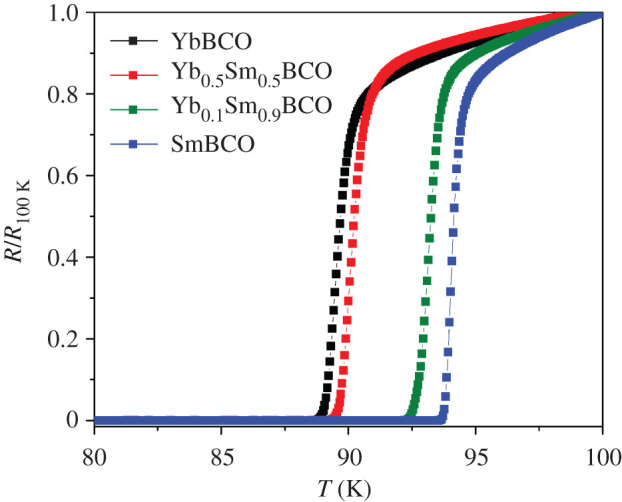
Normalized *R*(*T*) curves of Yb_1−*x*_Sm*_x_*Ba_2_Cu_3_O_7−_*_δ_* films grown with the parameters in table 1.

The *T*_c_ values, figure 2, vary between approximately 90.5 K for the pure YbBCO films and approximately 95 K for the pure SmBCO films. These values are similar to the ones reported in previous works for YbBCO [13–18] and SmBCO films [12,34–36] prepared by different techniques and also resemble previously reported values for YbSmBCO bulk samples [33]. The influence of Yb content on *T*_c_ is very remarkable. Already 10% of Yb (*x* = 0.9) reduces *T*_c_ by approximately 1 K. Moreover, for *x* = 0.5, the *T*_c_ of approximately 91 K is very similar to the pure YbBCO value. Thus, *T*_c_ is not following a linear dependence as reported in, for example, [13] for the case of Y_1−*x*_Gd*_x_*Ba_2_Cu_3_O_7−_*_δ_* films. The films with *x* = 0.5 have the largest Δ*T*_c_ of approximately 1.3 K, while in the rest of the films Δ*T*_c_ is approximately 1 K.

**Figure 3.**
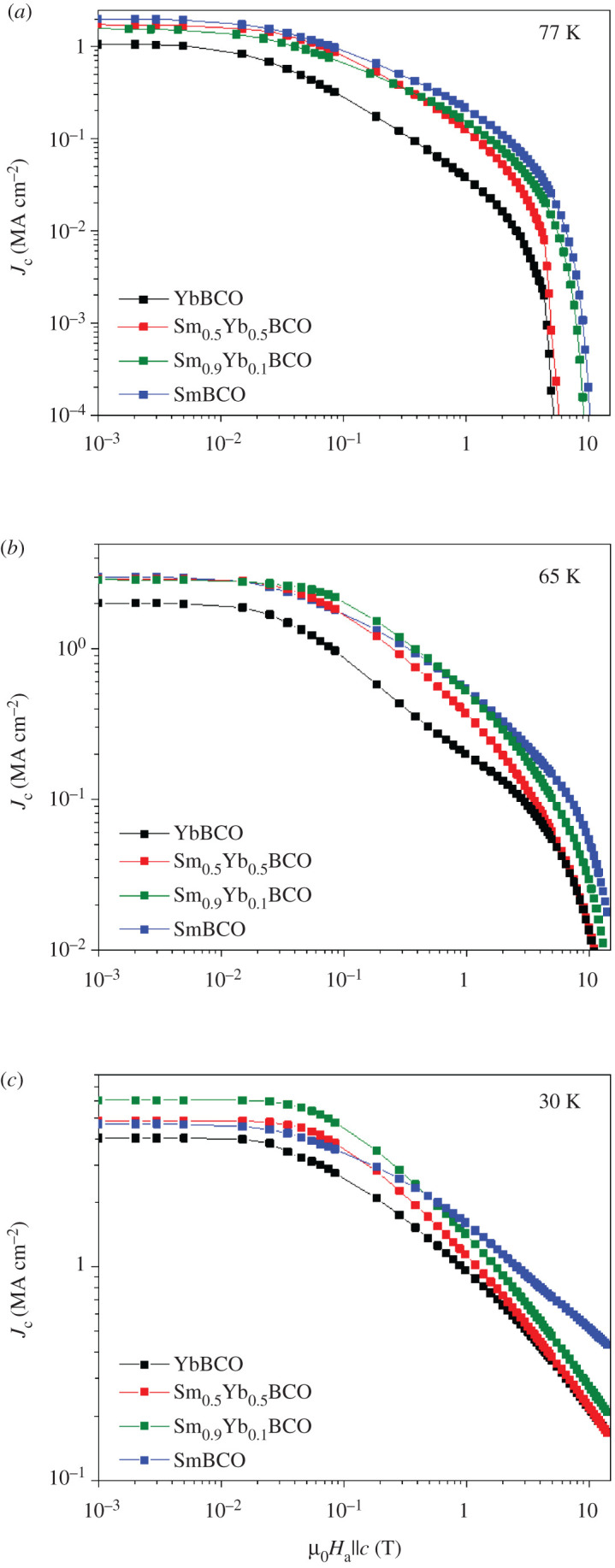
*J*_c_ dependence on magnetic field of Yb_1−*x*_Sm*_x_*Ba_2_Cu_3_O_7−_*_δ_* films at (*a*) 77, (*b*) 65 and (*c*) 30 K.

The magnetic field dependences of *J*_c_ at three different temperatures are compared in figure 3. $J_{c}^{\mathrm{sf}}$ at 77 K varies between approximately 1 MA cm^−2^ for pure YbBCO and approximately 2 MA cm^−2^ for pure SmBCO with the films with *x* = 0.5 and *x* = 0.9 in between. Although these values seem to be quite modest, they are among the largest published for YbBCO [13–18] and also SmBCO films [12,34–36] grown by different techniques. However, at lower temperatures, the mixed films start to compete with the pure phases. At 65 K, $J_{c}^{\mathrm{sf}}$ increases to the range approximately 3 MA cm^−2^ for the Sm-containing films and approximately 2 MA cm^−2^ for the pure YbBCO film. At 30 K, the film with *x* = 0.9 presents the largest $J_{c}^{\mathrm{sf}}$ of approximately 6 MA cm^−2^ followed by the two other Sm-containing samples with $J_{c}^{\mathrm{sf}}$ approximately 5 MA cm^−2^ and, again, the pure YbBCO film has the lowest value of approximately 4 MA cm^−2^. This behaviour is a combined effect of *T*_c_, microstructural defects and additional pinning due to *RE* mixing. The fact that at low temperatures, the mixed films have larger $J_{c}^{\mathrm{sf}}$ does, however, not mean that the in-field values are higher. At 65 and 30 K, the mixed films show larger *J*_c_ values at low fields but smaller values than SmBCO above a certain cross-over magnetic field. This cross-over depends both on *x* and temperature and is related to the fact that the values of the exponent *α* (*J*_c_ ∼ *B*^−^*^α^*) are larger for the mixed films or in other words to opposing trends for $J_{c}^{\mathrm{sf}}$ and irreversibility field (*H*_irr_). The same behaviour of mixed films with different combinations of *RE* ions was observed by MacManus-Driscoll *et al.* at 77 K [19]. In that work, this effect of enhancing the pinning at low fields was attributed to the random point-like disorder in those films, namely random displacements of oxygen ions due to the ion size variance. This could also be the explanation for the behaviour observed in our films.

**Figure 4.**
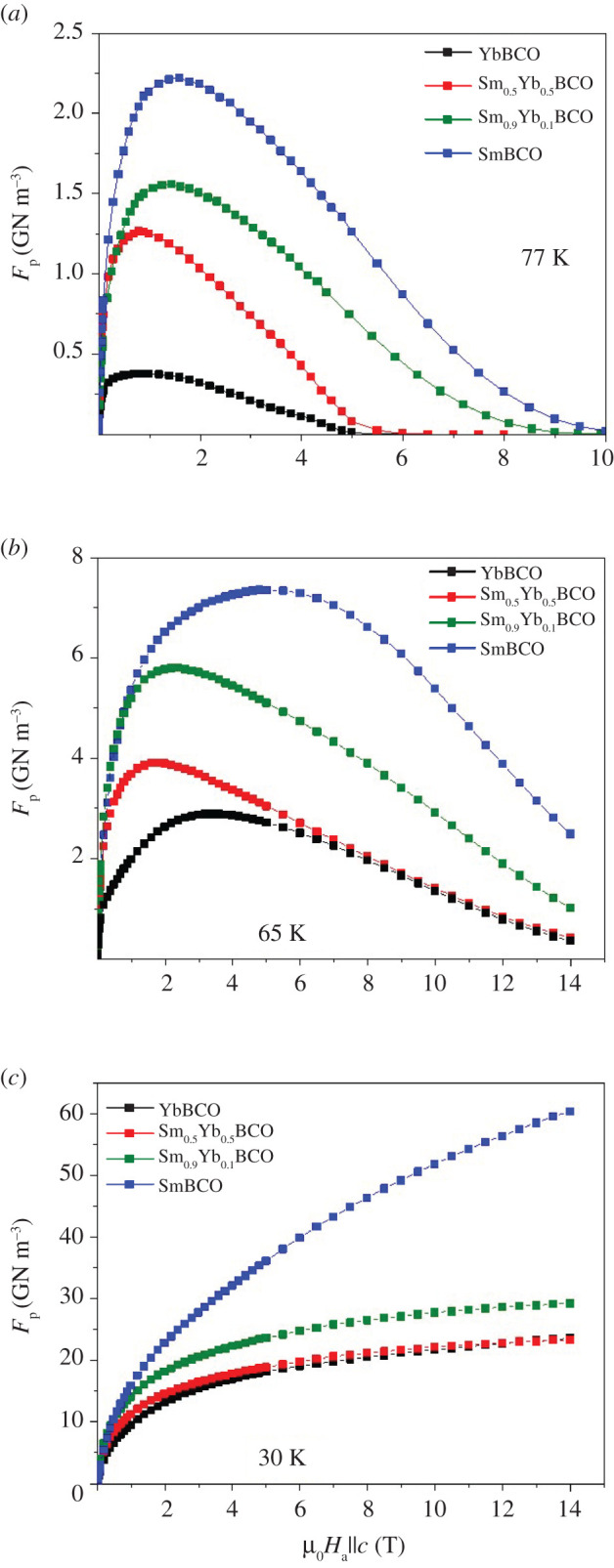
*F*_p_ dependence on magnetic field of Yb_1−*x*_Sm*_x_*Ba_2_Cu_3_O_7−_*_δ_* films at (*a*) 77, (*b*) 65 and (*c*) 30 K.

Figure 4 displays the magnetic field dependence of the pinning force density (*F*_p_) of the YbSmBCO films. The largest absolute *F*_p_ values belong to the pure SmBCO film at all temperatures: approximately 2.2 (at 1.6 T), approximately 7.4 (at 5.1 T) and greater than 60 GN m^−3^ at 77, 65 and 30 K, respectively. Due to the larger accommodation field H*, defined as *J*_c_(H*) = 0.9 $J_{c}^{\mathrm{sf}}$, there are narrow low-field regions at 65 and 30 K where *J*_c_ and hence *F*_p_ of the mixed films is larger. However, the maximum values of these films are lower than for the SmBCO film, e.g. approximately 1.5 (at 1.4 T), approximately 5.7 (at 2.3 T) and approximately 29.3 GN m^−3^ at 77 K, 65 K and 30 K, respectively, for *x* = 0.9.

## 4. Conclusion

The main objective of this work was to study the superconducting properties of *RE*BCO films with a mixture of *RE* ions with large difference in ion size, in particular Sm^3+^ and Yb^3+^. Despite the challenges associated with the ion size itself (Ba^2+^ substitution with Sm^3+^ and ion vacancies with Yb^3+^) and with the large difference in the optimum crystallization temperature between both pure compounds, it was possible to synthesize superconducting Yb_1−*x*_Sm*_x_*Ba_2_Cu_3_O_7−_*_δ_* (YbSmBCO) films on LAO substrates by CSD using ELF solutions. The *T*_c_ values are largely influenced by the presence of Yb^3+^ ions. The *J*_c_ values at low fields, especially at low temperatures, increase by the use of mixed phases but are inferior to values of pure compounds at high fields.

## Supplementary Material

Reviewer comments

## Data accessibility

Data supporting this study are available from the Dryad Digital Repository: https://doi.org/10.5061/dryad.jsxksn06p [37].
