# Supplementary material for: REBCO mixtures with large difference in rare-earth ion size: superconducting properties of chemical solution deposition-grown Yb1−xSmxBa2Cu3O7−δ films
Source: R Soc Open Sci. 2020 Nov 18;7(11):201257. doi: 10.1098/rsos.201257 (PMC7735353; doi:10.1098/rsos.201257)
Supplement: Reviewer comments [file rsos201257_review_history.pdf]

THE ROYAL SOCIETY
PUBLISHING

ROYAL SOCIETY
OPEN SCIENCE

*RE*BCO mixtures with large difference in rare-earth ion size: superconducting properties of chemical solution deposition-grown $Yb_{1-x}Sm_xBa_2Cu_3O_{7-\delta}$ films

Pablo Cayado, Minjuan Li, Manuela Erbe, Zhiyong Liu, Chuanbing Cai, Jens Hänisch and Bernhard Holzapfel

Article citation details
R. Soc. open sci. **7**: 201257.
http://dx.doi.org/10.1098/rsos.201257

Review timeline
Original submission: 27 July 2020
Revised submission: 22 September 2020
Final acceptance: 19 October 2020

Note: Reports are unedited and appear as submitted by the referee. The review history appears in chronological order.

Review History

RSOS-201257.R0 (Original submission)

Review form: Reviewer 1

Is the manuscript scientifically sound in its present form?
No

Are the interpretations and conclusions justified by the results?
Yes

Is the language acceptable?
No

Do you have any ethical concerns with this paper?
No

Have you any concerns about statistical analyses in this paper?
No

Reports © 2020 The Reviewers; Decision Letters © 2020 The Reviewers and Editors; Responses © 2020 The Reviewers, Editors and Authors. Published by the Royal Society under the terms of the Creative Commons Attribution License http://creativecommons.org/licenses/by/4.0/, which permits unrestricted use, provided the original author and source are credited

Recommendation?
Major revision is needed (please make suggestions in comments)

Comments to the Author(s)
The manuscript report on REBCO mixtures with large difference in rare-earth ion size: superconducting properties of chemical solution deposition grown Yb1-xSmxBa2Cu3O7-□ films. The author studied the superconducting properties of chemical solution deposition grown Yb1-xSmxBa2Cu3O7-□ films. The paper reports enough results but need to be rewritten. After going through the paper and literature of the same topic the paper needs major revision to publish in this journal. Some of the points are given below that author might consider to increase the intensity of the paper.

1. The abstract needs to rewritten.
2. The English must be improved. There are some grammatical and typos errors that the author must revise them.
3. The introduction of the manuscript should be specific which includes the significance of the materials, literature survey, advantages of the deposition technique over other techniques.
4. Authors should include SEM / TEM, AFM micrograph to observe the change in morphology and thickness measurement with the addition of Sm.
5. The author should highlight their unique and novelty compared with other kinds of films.

Review form: Reviewer 2

Is the manuscript scientifically sound in its present form?
Yes

Are the interpretations and conclusions justified by the results?
No

Is the language acceptable?
Yes

Do you have any ethical concerns with this paper?
No

Have you any concerns about statistical analyses in this paper?
No

Recommendation?
Major revision is needed (please make suggestions in comments)

Comments to the Author(s)
The study entitled, "REBCO mixtures with large difference in rare-earth ion size: superconducting properties of CSD grown Yb1- xSmxBa2Cu3O7- δ films", focused on the effect of the RE ions with large difference in ion size on the superconducting properties of REBCO films. The results are interesting, but are not organized and presented in a good manner. However, the manuscript should be considerably improved following items below with an English correction and can be accepted for publication after MAJOR REVISIONS:

Define TFA.
Page 2, line 39: what is the specific application mentioned in the text?
Introduction

Page 2, line 51: “the second-generation (2G) high-Tc superconducting tapes and the Coated Conductors (CCs)”

Check the presentation of the references, Example: [1-3] instead of [1]-[3].

Page 2, line 56: “Their high critical current densities, also in high magnetic fields”, I think “also” in not the right word to use in this sentence.

Page 3, line 4: You should mention why YBCO is the best among REBCO in order to compare your results with such phase.

Page 3, line 24: the sentence is written in a wrong way:

“In particular, the syntheses of SmBCO and YbBCO films are probably two of the most challenging ones among the REBCO phases because the ion size of both compounds are almost at the superior and inferior ends of the line of possible lanthanide candidates.”

Page 3, line 32: The synthesis ‘difficulties must be added briefly.

The motivation should be clearer and the literature review is not presented well.

Page 3, line 29: Please define TFA-MOD route.

Page 3, line 49: please re-write the procedure in a better way.

Section 3.1: while reading the results, I tried to understand the physical meaning of the context. Unfortunately, the whole paragraph describes the curves in figure 1. Please improve our discussion.

Page 6: you didn’t mention how you determined Tc.

The enhancement of Tc is due to what?

Page 6: what is the physical indication of ΔTc

Page 7, line 50: how did you determine the pinning force Fp?

Page 7, improve your discussions please.

Decision letter (RSOS-201257.R0)

We hope you are keeping well at this difficult and unusual time. We continue to value your support of the journal in these challenging circumstances. If Royal Society Open Science can assist you at all, please don't hesitate to let us know at the email address below.

Dear Dr CAYADO:

Title: REBCO mixtures with large difference in rare-earth ion size: superconducting properties of CSD grown Yb1-xSmxBa2Cu3O7- δ films
Manuscript ID: RSOS-201257

Thank you for your submission to Royal Society Open Science. The chemistry content of Royal Society Open Science is published in collaboration with the Royal Society of Chemistry.

The editor assigned to your manuscript has now received comments from reviewers. We would like you to revise your paper in accordance with the referee and Subject Editor suggestions which can be found below (not including confidential reports to the Editor). Please note this decision does not guarantee eventual acceptance.

Please submit your revised paper before 04-Oct-2020. Please note that the revision deadline will expire at 00.00am on this date. If we do not hear from you within this time then it will be assumed that the paper has been withdrawn. In exceptional circumstances, extensions may be possible if agreed with the Editorial Office in advance. We do not allow multiple rounds of revision so we urge you to make every effort to fully address all of the comments at this stage. If deemed necessary by the Editors, your manuscript will be sent back to one or more of the original reviewers for assessment. If the original reviewers are not available we may invite new reviewers.

To revise your manuscript, log into http://mc.manuscriptcentral.com/rsos and enter your Author Centre, where you will find your manuscript title listed under "Manuscripts with Decisions." Under "Actions," click on "Create a Revision." Your manuscript number has been appended to denote a revision. Revise your manuscript and upload a new version through your Author Centre.

When submitting your revised manuscript, you must respond to the comments made by the referees and upload a file "Response to Referees" in "Section 6 - File Upload". Please use this to document how you have responded to the comments, and the adjustments you have made. In order to expedite the processing of the revised manuscript, please be as specific as possible in your response.

Once again, thank you for submitting your manuscript to Royal Society Open Science and I look forward to receiving your revision. If you have any questions at all, please do not hesitate to get in touch.

Yours sincerely,
Dr Laura Smith
Publishing Editor, Journals

Royal Society of Chemistry
Thomas Graham House
Science Park, Milton Road
Cambridge, CB4 0WF
Royal Society Open Science - Chemistry Editorial Office

On behalf of the Subject Editor Professor Anthony Stace and the Associate Editor Dr Dattatray Late.

****

RSC Associate Editor:
Comments to the Author:
The work is of interest and I suggest authors to revise the manuscript as per reviewer's suggestions. Major Revision.

RSC Subject Editor:
Comments to the Author:
(There are no comments.)

****

Reviewers' Comments to Author:
Reviewer: 1

Comments to the Author(s)
The manuscript report on REBCO mixtures with large difference in rare-earth ion size: superconducting properties of chemical solution deposition grown Yb1-xSmxBa2Cu3O7-□ films. The author studied the superconducting properties of chemical solution deposition grown Yb1-xSmxBa2Cu3O7-□ films. The paper reports enough results but need to be rewritten. After going through the paper and literature of the same topic the paper needs major revision to publish in this journal. Some of the points are given below that author might consider to increase the intensity of the paper.
1. The abstract needs to rewritten.
2. The English must be improved. There are some grammatical and typos errors that the author must revise them.

3. The introduction of the manuscript should be specific which includes the significance of the materials, literature survey, advantages of the deposition technique over other techniques.
4. Authors should include SEM / TEM, AFM micrograph to observe the change in morphology and thickness measurement with the addition of Sm.
5. The author should highlight their unique and novelty compared with other kinds of films.

Reviewer: 2

Comments to the Author(s)
The study entitled, "REBCO mixtures with large difference in rare-earth ion size: superconducting properties of CSD grown Yb1- xSmxBa2Cu3O7- δ films", focused on the effect of the RE ions with large difference in ion size on the superconducting properties of REBCO films. The results are interesting, but are not organized and presented in a good manner. However, the manuscript should be considerably improved following items below with an English correction and can be accepted for publication after MAJOR REVISIONS:
Define TFA.
Page 2, line 39: what is the specific application mentioned in the text?
Introduction
Page 2, line 51: "the second-generation (2G) high-Tc superconducting tapes and the Coated Conductors (CCs)"
Check the presentation of the references, Example: [1-3] instead of [1]-[3].
Page 2, line 56: "Their high critical current densities, also in high magnetic fields", I think "also" in not the right word to use in this sentence.
Page 3, line 4: You should mention why YBCO is the best among REBCO in order to compare your results with such phase.
Page 3, line 24: the sentence is written in a wrong way:
"In particular, the syntheses of SmBCO and YbBCO films are probably two of the most challenging ones among the REBCO phases because the ion size of both compounds are almost at the superior and inferior ends of the line of possible lanthanide candidates."
Page 3, line 32: The synthesis 'difficulties must be added briefly.
The motivation should be clearer and the literature review is not presented well.
Page 3, line 29: Please define TFA-MOD route.
Page 3, line 49: please re-write the procedure in a better way.
Section 3.1: while reading the results, I tried to understand the physical meaning of the context. Unfortunately, the whole paragraph describes the curves in figure 1. Please improve our discussion.
Page 6: you didn't mention how you determined Tc.
The enhancement of Tc is due to what?
Page 6: what is the physical indication of Δ Tc
Page 7, line 50: how did you determine the pinning force Fp?
Page 7, improve your discussions please.

Author's Response to Decision Letter for (RSOS-201257.R0)

See Appendix A.

RSOS-201257.R1 (Revision)

Review form: Reviewer 1

Is the manuscript scientifically sound in its present form?
Yes

Are the interpretations and conclusions justified by the results?
Yes

Is the language acceptable?
Yes

Do you have any ethical concerns with this paper?
No

Have you any concerns about statistical analyses in this paper?
No

Recommendation?
Accept as is

Comments to the Author(s)
After the suggested modification, the manuscript is now acceptable for the publication.

Review form: Reviewer 2

Is the manuscript scientifically sound in its present form?
Yes

Are the interpretations and conclusions justified by the results?
Yes

Is the language acceptable?
Yes

Do you have any ethical concerns with this paper?
No

Have you any concerns about statistical analyses in this paper?
No

Recommendation?
Accept as is

Comments to the Author(s)
The paper in its corrected form can be accepted.

Decision letter (RSOS-201257.R1)

We hope you are keeping well at this difficult and unusual time. We continue to value your support of the journal in these challenging circumstances. If Royal Society Open Science can assist you at all, please don't hesitate to let us know at the email address below.

Dear Dr CAYADO:

Title: REBCO mixtures with large difference in rare-earth ion size: superconducting properties of CSD grown Yb1-xSmxBa2Cu3O7-δ films
Manuscript ID: RSOS-201257.R1

It is a pleasure to accept your manuscript in its current form for publication in Royal Society Open Science. The chemistry content of Royal Society Open Science is published in collaboration with the Royal Society of Chemistry.

The comments of the reviewer(s) who reviewed your manuscript are included at the end of this email.

Thank you for your fine contribution. On behalf of the Editors of Royal Society Open Science and the Royal Society of Chemistry, I look forward to your continued contributions to the Journal.

Yours sincerely,
Dr Laura Smith
Publishing Editor, Journals

Royal Society of Chemistry
Thomas Graham House
Science Park, Milton Road
Cambridge, CB4 0WF
Royal Society Open Science - Chemistry Editorial Office

On behalf of the Subject Editor Professor Anthony Stace and the Associate Editor Dr Dattatray Late.

****

RSC Associate Editor:
Comments to the Author:
(There are no comments.)

RSC Subject Editor:
Comments to the Author:
(There are no comments.)

****

Reviewer(s)' Comments to Author:
Reviewer: 1
Comments to the Author(s)
After the suggested modification, the manuscript is now acceptable for the publication.

Reviewer: 2
Comments to the Author(s)
The paper in its corrected form can be accepted.

Appendix A

We thank the editorial board members and reviewers for their comments and suggestions which we have considered for improving the manuscript.

RSC Associate Editor:
Comments to the Author:
The work is of interest and I suggest authors to revise the manuscript as per reviewer's suggestions. Major Revision.

We thank the associate editor for the positive assessment of our paper. We have revised our manuscript following the reviewers' suggestions.

Reviewers' Comments to Author:
Reviewer: 1

Comments to the Author(s)
The manuscript report on REBCO mixtures with large difference in rare-earth ion size: superconducting properties of chemical solution deposition grown Yb1-xSmxBa2Cu3O7-δ films. The author studied the superconducting properties of chemical solution deposition grown Yb1-xSmxBa2Cu3O7-δ films. The paper reports enough results but need to be rewritten. After going through the paper and literature of the same topic the paper needs major revision to publish in this journal. Some of the points are given below that author might consider to increase the intensity of the paper.

1. The abstract needs to rewritten.

We thank the reviewer for the suggestion. We have checked and rewritten the abstract following the proposed advice.

2. The English must be improved. There are some grammatical and typos errors that the author must revise them.

We thank the reviewer for the suggestion. We have revised the text and corrected all the grammatical and orthographic errors that we have found.

3. The introduction of the manuscript should be specific which includes the significance of the materials, literature survey, advantages of the deposition technique over other techniques.

We thank the reviewer for the suggestion. We have revised the introduction and adapted it to as proposed by the reviewer.

4. Authors should include SEM / TEM, AFM micrograph to observe the change in morphology and thickness measurement with the addition of Sm.

Unfortunately, we have no access to a TEM to present such images but we think that they are not necessary since the aim of the work was to show how the superconducting properties change when mixing different amounts of very different in size RE ions. This is already shown in the paper.

Regarding the SEM and AFM, we have not included them because, on the one hand, we have already published images taken by these two pieces of equipment in our previous paper about pure SmBCO films (reference 12 in the manuscript) showing the surface morphology and the thickness of the films and, on the other hand, because the use of different Sm/Yb ratios do not change significantly the surface morphology or the thickness. Therefore, the images published in reference 12 are a good example for the films on this work and there is no need to show them here again.

5. The author should highlight their unique and novelty compared with other kinds of films.

The novelty of this work is the preparation of $Yb_{1-x}Sm_xBa_2Cu_3O_{7-\delta}$ films by CSD for the first time, a type of films that mix RE ions with a large difference in ion size. Moreover, we have investigated the properties of these films when varying the Sm/Yb ratio, something that is done for the first time due to the extremely difficult synthesis process of these films. This novelty was included in abstract and conclusions: "The main objective of this work was to study the superconducting properties of *RE*BCO films with a mixture of *RE* ions with large difference in ion size, in particular Sm^{3+} and Yb^{3+}." It is further mentioned in section 3.2: "This has been the first time that ELF solutions were used to successfully grow this type of films, and one of the first to study this rare-earth combination at all [33]."

Reviewer: 2

Comments to the Author(s)

The study entitled, "REBCO mixtures with large difference in rare-earth ion size: superconducting properties of CSD grown Yb1- xSmxBa2Cu3O7-δ films", focused on the effect of the RE ions with large difference in ion size on the superconducting properties of REBCO films. The results are interesting, but are not organized and presented in a good manner. However, the manuscript should be considerably improved following items below with an English correction and can be accepted for publication after MAJOR REVISIONS:

-Define TFA.

We have defined TFA in the text as: trifluoroacetate (TFA)

-Page 2, line 39: what is the specific application mentioned in the text?

The sentence in the abstract "…reveals the importance of selecting adequate *RE*BCO compounds according to the temperature and magnetic field region where a specific application operates." is not referring to any application in particular. Instead, it means that once one has an application in mind that requires the use of high temperature superconductors, in particular, of *RE*BCO compounds, one should be careful when selecting the specific *RE*BCO compound because their properties vary a lot from compound to compound and even more when mixed. Therefore, once you define the temperature and field range in which your particular application will operate, you can select the most appropriate *RE*BCO compound whose properties are optimum in the operation temperature and field range.

-Introduction. Page 2, line 51: "the second-generation (2G) high-Tc superconducting tapes and the Coated Conductors (CCs)" Check the presentation of the references, Example: [1-3] instead of [1]-[3].

We have changed the presentation of the references following the reviewer's suggestion.

-Page 2, line 56: "Their high critical current densities, also in high magnetic fields", I think "also" in not the right word to use in this sentence.

We have revised the sentence and changed it to "even".

-Page 3, line 4: You should mention why YBCO is the best among REBCO in order to compare your results with such phase.

We have modified the original sentence to "…which forms $YBa_2Cu_3O_{7-\delta}$ (YBCO), the best-studied *RE*BCO compound.". This sentence does not mean that the YBCO is the best compound among the *RE*BCO but it means that is the most studied one, probably because it was the first *RE*BCO compound to be discovered and is relatively stable and easy to prepare.

-Page 3, line 24: the sentence is written in a wrong way: "In particular, the syntheses of SmBCO and YbBCO films are probably two of the most challenging ones among the REBCO phases because the ion size of both compounds are almost at the superior and inferior ends of the line of possible lanthanide candidates."

We thank the referee for this suggestion and changed the sentence for more clarity to "In particular, SmBCO and YbBCO are probably two of the most challenging *RE*BCO phases to prepare as high-quality films or bulks because the *RE* ion sizes of these two compounds are almost at the lower and the upper end of all possible lanthanide candidates.

-Page 3, line 32: The synthesis 'difficulties must be added briefly. The motivation should be clearer and the literature review is not presented well.

The synthesis difficulties are already included in the manuscript in the following piece of text: "However, the synthesis of these compounds is occasionally more complicated than for YBCO. On the one hand, large *RE* ions, like Nd^{3+} or Sm^{3+}, tend to partially replace the Ba^{2+} ions and, on the other hand, small RE^{3+} ions, such as Yb^{3+} and Lu^{3+}, do not fit satisfactorily in their lattice site generating vacancies. Both facts cause a drastic decrease of the *RE*BCO phase stability and superconducting properties [9 –11]."

-Page 3, line 29: Please define TFA-MOD route.

TFA-MOD route is now defined in the manuscript.

-Page 3, line 49: please re-write the procedure in a better way.

We have improved the language of the experimental section following the reviewer advise and added details for pyrolysis and growth for better clarity. We believe that further information

should be taken from ref. 12 and references therein since that is not the main point of this publication.

-Section 3.1: while reading the results, I tried to understand the physical meaning of the context. Unfortunately, the whole paragraph describes the curves in figure 1. Please improve our discussion.

Figure 1 serves as visual guidance through that section for faster reading and understanding of that matter. Therefore, it is not surprising that the paragraph describes (and explains!) that figure. We improved the language of that section for better clarity. Besides that, it contains already all information there is, namely the similarities and differences to RE mixtures of rather similar size. The microscopic or chemical reasons for the much smaller temperature windows are unknown to date.

-Page 6: you didn't mention how you determined Tc. The enhancement of Tc is due to what?

As it is written in the experimental section ("The critical temperature T_c ($T_{c,90}$, i.e., the temperature at which the resistance is 90 % of the value above the transition), ΔT_c ($T_{c,90} - T_{c,10}$), $J_c(B)$ (via $V(I)$ curves with 1 μV/cm criterion) and the J_c anisotropy of the films were studied with a 14-T *Quantum Design* Physical Property Measurement System (PPMS) in 4-point geometry."), the T_c was measure by transport in a PPMS. We measured the the temperature dependence of the resistance know as R(T) curve. T_c was then extracted as the temperature at which the resistance is 90 % of the value just above the transition, usually at 95 K or 100 K.

The values of T_c vary because we are changing the ratio Sm/Yb in the films and this affects the final T_c of the films. The pure SmBCO films show a T_c ~ 95 K while the pure YbBCO films ~ 90 K. Varying the amount of both compounds in the final mixture, we reach different values of T_c.

-Page 6: what is the physical indication of ΔTc

As explained in the experimental section, the ΔT_c is calculated from the R(T) curves simply by subtracting $T_{c,10}$ from $T_{c,90}$ (see previous comment for the meaning of $T_{c,90}$ and $T_{c,10}$). This parameter is related with the homogeneity of the films in terms of composition. A narrow ΔT_c, i.e. low values, means that most parts of the film have the same T_c value, i.e. almost the same composition, while a broad ΔT_c means that there is a spatial distribution of Tc values, i.e. compositional inhomogeneity.

-Page 7, line 50: how did you determine the pinning force Fp?

We have included an explanation of how the pinning force is calculated in the new version of the manuscript. The F_p is simply calculated by multiplying the J_c by the magnetic field, B.

-Page 7, improve your discussions please.

We have improved the language of section 3 for better clarity. Besides that, we believe it contains all accessible information there is, especially for a fast-track first-of-its-kind publication as intended.

We again thank both referees for their critical reading and valuable inputs and hope our changes and improvements meet their expectations.

In the name of all co-authors
with best regards,

Pablo Cayado
